# Counter Irritants in Sciatica

**Published:** 1897-11-27

**Authors:** 


### COUNTER-IRRITANTS IN SCIATICA.

An ingenious patient, whose intelligence was far greater than his knowledge, has discovered that sciatica can be cured, or, rather, that the pain of sciatica can be relieved, by painting the skin over the nerve with hydrochloric acid. It appears that the patient had been treated in a hospital in Algeria by the hypodermic injection of salt and water. The effect was not encouraging. This lack of success the patient attributed to the salt not being strong enough; and imagining that spirit of salt would be stronger than the ordinary article he obtained some of this material, which we hardly need say is hydrochloric acid, and painted it on the painful part. The result was that he was rapidly relieved. This wonderful fact he imparted to his doctor, who has since tried the remedy in many cases with much success, and now a thesis has been written upon it by Dr. Gennatas, of Montpellier. The matter has got into the *Semaine Medicale*, and thence into the *Lancet*, which tells the tale and describes the process. Strong hydrochloric acid is painted over the painful part of the nerve, three or four coats being applied, and the limb is then enveloped in a cotton-wool dressing. Severe smarting is felt for a time, the skin becomes reddened and hot, and sometimes bullæ form. Usually the patient feels better even after a single sitting. The process may be repeated after twenty-four hours, but care must be taken to avoid any bullæ which may have been formed, and not to apply the acid sufficiently freely to cause sloughing. Of course, counter-irritation is a very, very old mode of treatment in sciatica, and a host of different substances have been used for the purpose. If counter-irritation is to be used the great thing is to do it *con amore*. It is no use being over tender about it. Blisters, Corrigan's button, iodine, strong liniments, and varying acids have all been used, and all with considerable success—with much greater success, however, in some hands than in others, which no doubt has been due to the varying energy with which they have been employed. This little paragraph in the *Lancet* must not lead us to imagine that some discovery has been made. It is as old as the hills; in fact, the treatment of neuralgia by acid of another sort, the acetic, has been elevated to the dignity of an "opathy," and we believe that in various parts of the country professors of "acetopathy" are still to be found. It may fairly be admitted that we know but little as to the mode of action of counter-irritants, but this we do know, that they do not act unless they irritate. As to the substance which is used for the purpose, that is probably to a large extent a matter of indifference if we bear in mind these two points, that for pure neuralgia, if there is such a thing, the more transient the irritation the better, while for deep inflammatory changes a certain continuity of action seems desirable. It is not that some substances do more good than others, but that some do less harm; and among these less harmful irritants iodine, acetic acid, and hydrochloric acid may fairly be placed. The "acetopathists," we believe, usually employ their acid hot as well as strong.

